# Novel 4D radiomics applied to dynamic FES PET images to improve prediction of breast cancer response to ER-targeted therapy

**DOI:** 10.1007/s00259-025-07570-y

**Published:** 2025-11-23

**Authors:** Andrew William Chen, Carla R. Zeballos Torrez, Lanell M. Peterson, Mark Muzi, Jennifer M. Specht, Eric A. Cohen, Hannah M. Linden, Despina Kontos, David A. Mankoff

**Affiliations:** 1 Department of Radiology, Columbia University Irving Medical Center, Columbia University, New York, NY USA; 2 Department of Radiology, Perelman School of Medicine, University of Pennsylvania, 1 Donner Building, 3400 Spruce Street, Philadelphia, PA 19104 USA; 3 Department of Radiology, University of Washington, Seattle, WA USA; 4 Department of Hematology & Oncology, University of Washington, Seattle, WA USA

**Keywords:** Fluoroestradiol, Radiomics, Dynamic PET, Metastatic breast cancer, Endocrine therapy

## Abstract

**Purpose:**

[^18^F] fluoroestradiol (FES) is an FDA-approved tracer that measures functional estrogen receptor (ER) expression and can estimate the likelihood of response to ER-targeted therapy. In this exploratory analysis, we tested a novel radiomics based analysis of dynamic volumetric FES PET images to predict outcomes in patients with metastatic ER positive breast cancer treated with endocrine therapy.

**Methods:**

We utilized the Rad-Fit method, previously tested in an FDG PET data set, to identify and characterize intratumor subregions of heterogeneous time-activity through an unsupervised clustering approach. A scaled silhouette score was implemented to determine the optimal number of intratumor subregions on a per-tumor basis. Summary statistics of sum of squared error (SSE) and distance between sub regions as well as the total number of intratumor subregions were used to build prognostic models of overall survival (OS) and progression free survival (PFS). We employed Kaplan-Meyer analysis to determine model performance.

**Results:**

The radiomic phenotype differentiated between a high and low risk group for progression free survival (*C* = 0.67, *p* = 0.025) in the single tumor scenario. Radiomic features of subregion distance classified a high and low risk group for OS in a single tumor (*C* = 0.67, *p* = 0.008) and average tumor (*C* = 0.65, *p* = 0.017) scenario.

**Conclusions:**

In this exploratory study, 4D radiomic features extracted from dynamic FES PET images can improve the prediction of outcomes in metastatic ER positive breast cancer. Metrics of tumor subregion distance and radiomic phenotype appear to perform as the best radiomic predictors for risk stratification of OS and PFS respectively by potentially reflecting characteristics of the overall tumor heterogeneity in FES PET images.

Clinical trial number: not applicable.

**Supplementary Information:**

The online version contains supplementary material available at 10.1007/s00259-025-07570-y.

## Introduction

Breast cancer is the second leading cause of cancer death in all women [1]. Approximately 80% of all newly diagnosed breast cancers are hormone receptor (HR) positive (estrogen receptor (ER) positive and/or progesterone receptor (PR) positive), and have the best overall prognosis [2]. Patients with localized HR-positive disease have a 5-year survival rate of 99–100% compared to 34–46% for those with metastatic HR-positive breast cancer [2]. The use of ER-targeted therapies with or without CDK4/6 inhibitors is the most effective way of treating metastatic ER positive breast cancer [3, 4]. Approximately 13–20% of patients with breast cancer are HER2 positive [2, 5]; HER2 positive breast cancers were historically associated with an aggressive phenotype, poor survival outcomes, and decreased response to chemotherapy and endocrine therapy [6]. The development of HER2 targeted therapies has improved clinical outcomes [5, 7, 8] and improved survival for patients with metastatic HER2 positive breast cancer [9, 10]. Approximately half of HER2 positive breast cancers are ER and/or PR positive; the use of dual ER-HER2 targeted therapies in patients with ER positive/HER2 positive breast cancer demonstrates improved outcomes [11–14]. The implementation of dual HER2 blockade to ER-targeted therapy in this population, as demonstrated in the PERTAIN study, demonstrated statistically significant progression-free survival (PFS) improvement but no overall survival (OS) benefit [11]. While endocrine therapy and HER2-targeted agents improve survival, several mechanisms of acquired resistance, including intratumor heterogeneity [15–17], pose a challenge to treatment in the metastatic setting [18, 19].

The current practice of using tissue sampling of a selected site and assay for ER expression cannot assess the full burden of disease in metastatic breast cancer. Molecular imaging with PET/CT using radiotracer 16α-[^18^F]-fluoro-17β estradiol or ^18^F-fluoroestradial (FES) has been shown to provide an assessment of ER expression comparable to tissue sampling and IHC [20, 21] with high agreement between FES PET results and immunohistochemical ER status [22–24]. FES PET/CT has also been shown to predict response of metastatic breast cancer to endocrine therapy [21, 25, 26] and capture the heterogeneity of expression between sites of disease in metastatic breast cancer [20, 27]. FES is approved for use in the United States (™Cerianna) as an adjunct to biopsy in patients with ER-positive recurrent or metastatic breast cancer as a means to assess the variability and extent of ER expression [28–32].

In this study we applied the Rad-Fit method, a radiomics approach previously tested in an FDG data set, to FES PET/CT of patients with metastatic or recurrent ER-positive breast cancers treated with endocrine therapy to improve the prediction of outcomes. Radiomics can provide insights into tumor phenotype and interaction of the tumor with its microenvironment [33–35], thereby characterizing intratumor heterogeneity. Tumor heterogeneity in cancers is a well-established key prognostic and predictive factor [36, 37], associated with adverse outcomes [38–42], and may drive recurrence and therapy resistance [34, 38]. Extracted radiomic features characterizing tumor heterogeneity can be utilized as novel non-invasive prognostic biomarkers. In breast cancer, the application of radiomics to FDG PET/CT can be used to characterize tumor heterogeneity and predict axillary lymph node status [43–48], and predict the likelihood of response to treatment [49–53], and outcomes [51, 52, 54]. Most studies to date have focused on static imaging and measures of tracer uptake at a single time point. Dynamic PET imaging provides kinetic information [55] and can provide information relevant to response assessment [56, 57] and prognosis [58]. Members of our team have developed a functional 4D clustering approach for application to dynamic (4D) PET image data to characterize the radiomic functional intratumor heterogeneity (Rad-Fit) [58]. Regions of functional tumor heterogeneity (FTH) are determined by utilizing an unsupervised clustering approach on the voxels of 4D PET images, incorporating a Markov-Random field image segmentation method. This approach was shown to predict recurrence free survival using the FDG PET/CT images of a cohort of patients with histologically confirmed breast cancer [58].

In this exploratory study, we applied a 4D Rad-Fit approach to dynamic FES PET imaging studies, to assess predictive and prognostic 4D radiomic features for outcome in patients with metastatic ER positive cancer treated with endocrine therapy. This is the second application of the Rad-Fit radiomics method to dynamic whole body PET imaging.

## Materials & methods

### Study cohort

We investigated the role of these unique 4D radiomic features in characterizing the response to endocrine therapy in a historical FES PET dataset that was previously analyzed and reported using static FES uptake measures (SUV and qualitative uptake) and shown to predict response to endocrine therapy [26]. For this study, we used the imaging data from the original publication, however the clinical follow-up data on progression-free and overall survival was expanded in the time subsequent to the original publication. We further expand the analysis with our Rad-Fit method. Full follow-up data has been completed on all study participants and the dataset has been fully anonymized.

**Fig. 1:**
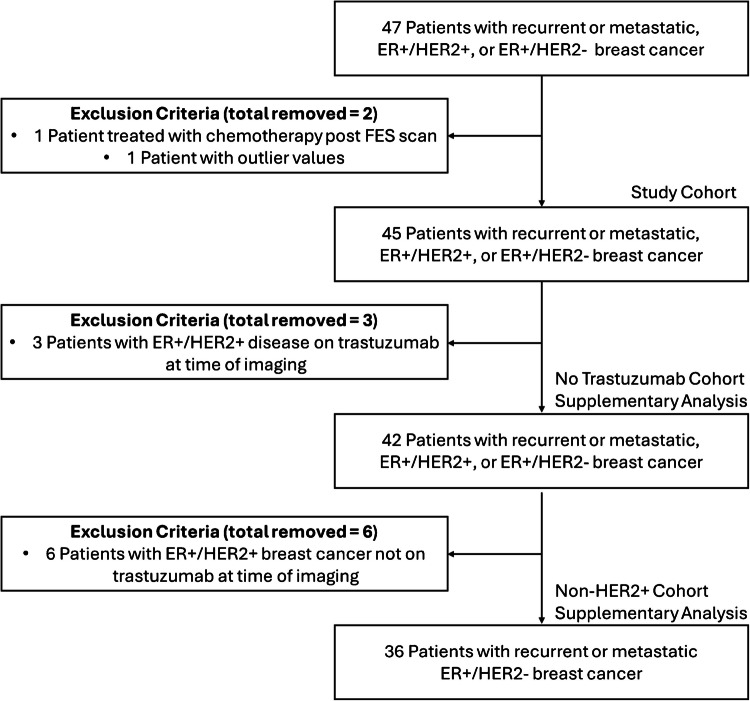
Chart of study population. The study cohort consists of 45 patients, with two additional subpopulations identified for supplementary analysis

The study cohort and additional subpopulations identified for supplemental analysis is shown in Fig. 1. Our final anonymized dataset consists of 45 patients from this study who underwent FES PET at the University of Washington Breast Cancer Specialty Center and had follow up to determine progression-free and overall survival. The original study was approved by the University of Washington (Seattle) IRB. Imaging was performed after obtaining informed consent and adhering to institutional IRB guidelines. To account for the heterogeneous tumor characteristics in our cohort, the inclusion of HER2 negative and HER2-overexpressing tumors (HER2 +), we analyzed two additional subpopulations; a subpopulation of 42 patients excluding three patients who were taking trastuzumab (Herceptin) at the time of imaging, and a subpopulation of 36 patients excluding six patients who had HER2 positive cancer. At the time of enrollment (1997–2003), treatment for HER2 positive breast cancer was in development. Trastuzumab was first approved in 1998 for metastatic HER2 positive breast cancer [59] with a subsequent phase III clinical trial published in 2001 [60] demonstrating the improved response rates and improvement in median overall survival for patients with metastatic HER2 positive breast cancer. Prior to the routine use of trastuzumab in HER2 positive tumors, patients were commonly treated with traditional chemotherapy regimens; currently, HER2 positive metastatic breast cancer patients have a multitude of options available with anti-HER2 therapies in combination with chemotherapy [61].

Imaging was performed after obtaining informed consent and adhering to institutional IRB guidelines. Briefly, the study patients had presented at the University of Washington Breast Cancer clinic with recurrent or metastatic breast cancer from ER-positive primary tumors confirmed by immunohistochemistry. All patients underwent endocrine treatment without cytotoxic chemotherapy or radiotherapy after FES PET imaging. Additional associated clinical variables are presented in supplementary Table S1.

Each patient underwent a 60-min FES PET scan centered at the most prominent site of disease as determined by standard staging studies (FDG PET, CT, bone scan) using a dynamic imaging regimen which contained volumes of 128 × 128 pixels × 35 slices at 24 imaging time points from injection to 60 min post-injection [26]. All patients enrolled had at least one disease site of at least 1.5 cm maximal dimension and patients with liver metastasis only were excluded as FES is cleared hepatically. Patients were enrolled in studies over a period of seven years. The minimum follow-up time for this cohort is nine months and the longest time to follow up is 17.5 years after the date of FES PET acquisition. Of the 45 patients only one has no recorded date of disease progression or death.

### 4D feature extraction

A 3D mask for each lesion was drawn from the 30–60 min summed images by radiologists (CZT and DM) blinded to patient outcome and clinical features and based on regions used in the original published analysis [26] using ITK-SNAP [62]. The 3D tumor region of each patient was applied to their dynamic FES PET image series to extract the 4D tumor region dynamic image data set. We then applied the Rad-Fit clustering approach to each segmented tumor voxel set [58].

Rad-Fit is a previously published algorithm which uses an unsupervised clustering approach incorporating Markov-Random field image segmentation to characterize the kinetic intratumor heterogeneity. In the implementation of Rad-Fit to this 4D FES PET dataset, the number of intratumor functional subregions was not pre-defined. Though the Rad-Fit algorithm was previously described [58], we provide a summary here as well as highlight some of the differences that are a result of the automated determination of the number of functional subregions individually in each tumor. The code used to implement the framework is available upon reasonable request. Relevant implementation details are provided herein to support reproducibility.

Each tumor was clustered into subregions based on the functional principal components (FPCs) of each voxel using the Rad-Fit method eleven times with the number of subregions increasing from two up to twelve subregions in each sequential clustering. A weighted silhouette score (*S*) was calculated (eq. [1]) on each of the eleven different segmentations generated for each tumor. This silhouette score was used to determine the optimal number of clusters for each tumor [63].

1 $$S= \left(\sum_{i}^{N}\left(\frac{{b}_{i}{-a}_{i}}{max({a}_{i},{b}_{i})}\right)/N\right)/\frac{min\left(C\right)}{max\left(C\right)}$$

*S* was calculated as the average silhouette score over all *N* voxels (eq. [1]) in the tumor where *a*_*i*_ is the average distance of the i^th^ point to all its other cluster members and *b*_*i*_ is the average of the minimum distance of the i^th^ point to points in all clusters for which it is not a member. *C* is the set of cluster sizes and *S* is scaled by the ratio of smallest to largest cluster size. Clusterings with singleton clusters, where any value in the set of cluster sizes *C* is one, are rejected from consideration. The nine 4D radiomic features extracted from each tumor were the total number of subregions, the mean, maximum, minimum, and standard deviation of the sum of squared errors (SSE) of voxel membership to the subregions and the mean, minimum, maximum and standard deviation of the distance between tumor subregion centers in FPC space. Rad-Fit segmentation and 4D radiomic feature extraction were performed in MATLAB R2021A (MathWorks, Natick, MA, USA). To further decrease dimensionality, we make use of a radiomic phenotype determined by unsupervised hierarchical clustering on the 4D feature set [65]. The number of significant clusters is interpreted as the number of intrinsic radiomic phenotypes in the cohort. This phenotype can be treated as an independent predictor.

### Predicting treatment outcomes

**Fig. 2:**
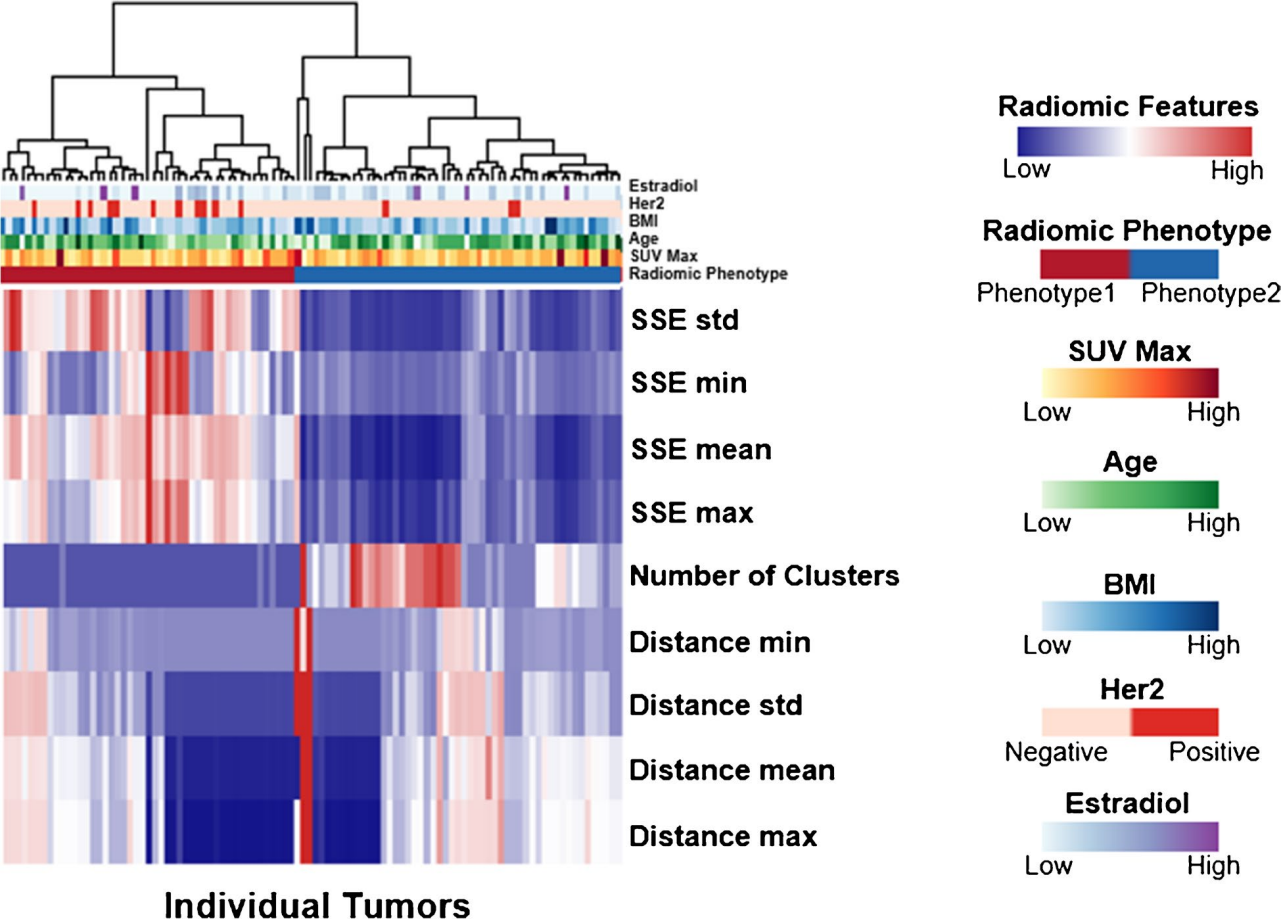
Heatmap of the 4D radiomic features of each tumor in the patient population (*n = *45) with other clinical variables used for prognostic modeling. The radiomic phenotypes which are a result of the unsupervised hierarchical clustering are shown at the top of the heatmap

In this study patients had one to three lesions labeled in the dynamic imaging FOV for analysis. The average of the 4D radiomic features across all tumors in an individual were used as well as a single tumor qualitatively judged to be the most prominent lesion from each patient by highest SUV. All statistical analysis was performed in R version 4.3.2 [64]. Z-score normalized radiomic features, radiomic phenotype, tumor SUVmax and clinical variables (age, BMI, estradiol levels, HER2 status) were used to build Cox proportional hazards (Cox-PH) models of OS and PFS, which were assessed primarily by medical record review. The mean OS time is 76.2 months with a minimum of 9 month and maximum of 243.4 months. The mean PFS time is 26.8 months with a minimum of 0.7 months and maximum of 243.4 months. Radiomic phenotypes (Fig. 2) were determined by performing unsupervised hierarchical clustering on the feature set [65]. The discriminatory power of the Cox-PH models was evaluated using the concordance test. Individual patients were categorized as being in the low or high-risk group depending on whether they were above or below the median hazard score and the significance of the risk groups was determined using a Kaplan–Meier (KM) plot.

After performing survival analysis using all radiomic features, we retained for subsequent analysis only those features with a Wald test *p* value < 0.05 indicating a statistically significant non-zero coefficient. We further refined our models by combining this subset of radiomic features in two ways 1) with the subset of clinical variables which are most significantly non-zero and 2) with SUVmax. We also compare to models built using only radiomic phenotype and SUVmax as well as a binary coding of SUVmax with a cutoff of 1.5 which was previously shown to have association with response to hormone therapy in work previously published with this dataset [26]. For all the models built we also tested the generalizability of these models using fivefold cross validation framework. A full list of model variables, HR, and 95% Cl, and Wald test *p-*values are available in the supplementary data (Tables S8-10).

## Results

**Fig. 3:**
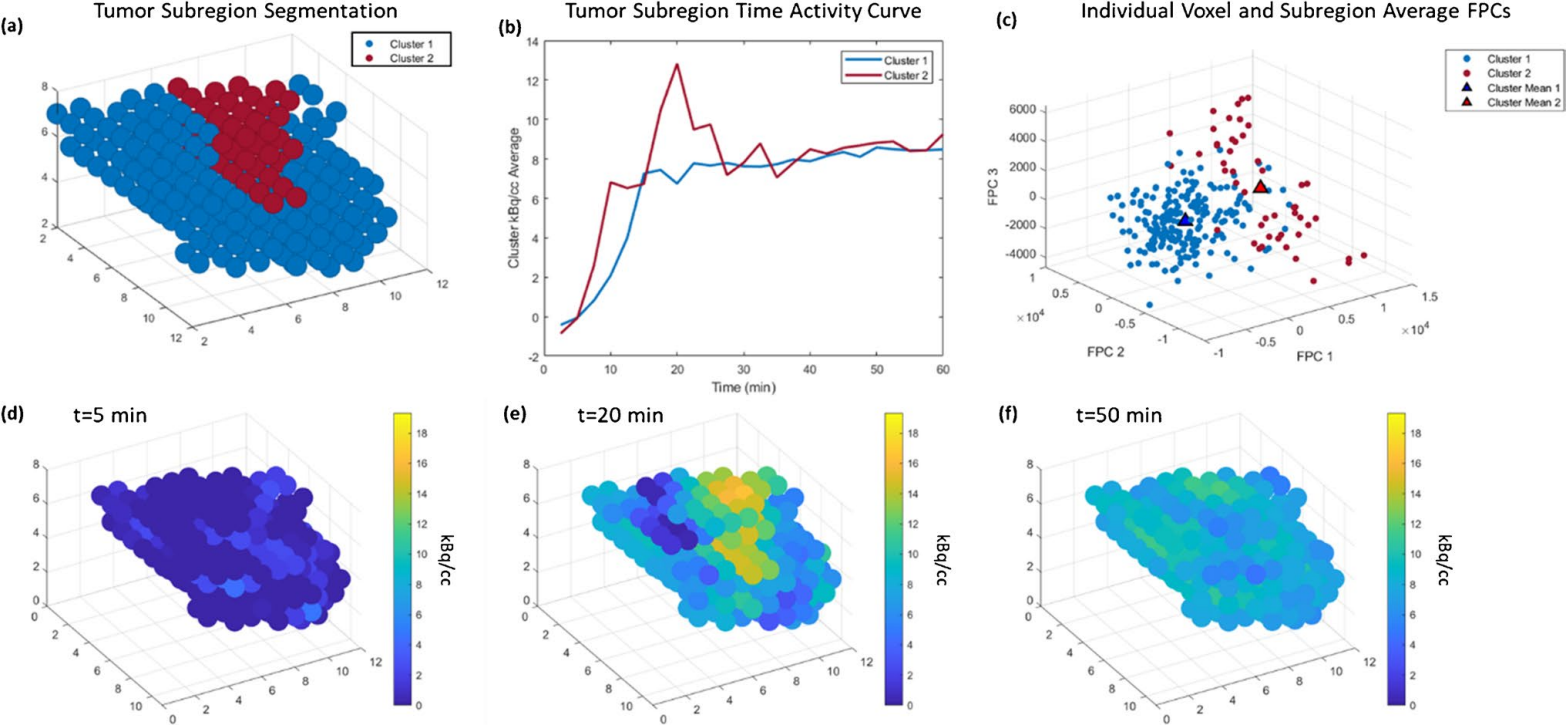
**a** An example segmentation of a tumor into two distinct subregions and **b** the time activity curve from each subregion. **c** Each voxel from each subregion is shown according to their first three FPCs with the centers of each cluster of points also shown. Tumor region intensity is shown at **d** 5 min is homogeneous, **e** at 20 min the intensity from one of the subregions is showing a much higher uptake, **f** at 50 min all regions reflect a more homogeneous intensity once again

A heatmap of the 4D radiomic features for each tumor from the cohort is shown in Fig. 2 separated by their intrinsic radiomic phenotype. Fifty-three tumors were members of phenotype one and forty-seven tumors were members of phenotype 2. Figure 3 shows an example of the FPC clustering for a case where the tumor was segmented into two distinct subregions (Fig. 3a). The summarized average uptake from each subregion (Fig. 3b) shows an initial equivalent uptake with one subregion quickly reaching plateau while the other subregion continues to increase to a higher peak before returning to the same plateau. Snapshots of the tumor volume over time reveal the regions resulting from FPC segmentation begin at equal intensity (Fig. 3d); one subregion reaches a higher average intensity towards the middle of the scan (Fig. 3e) and by the end of the scan both subregions are close to the same average intensity (Fig. 3f). An example of a tumor segmented into three subregions is shown in supplementary Fig. S2.

**Fig. 4:**
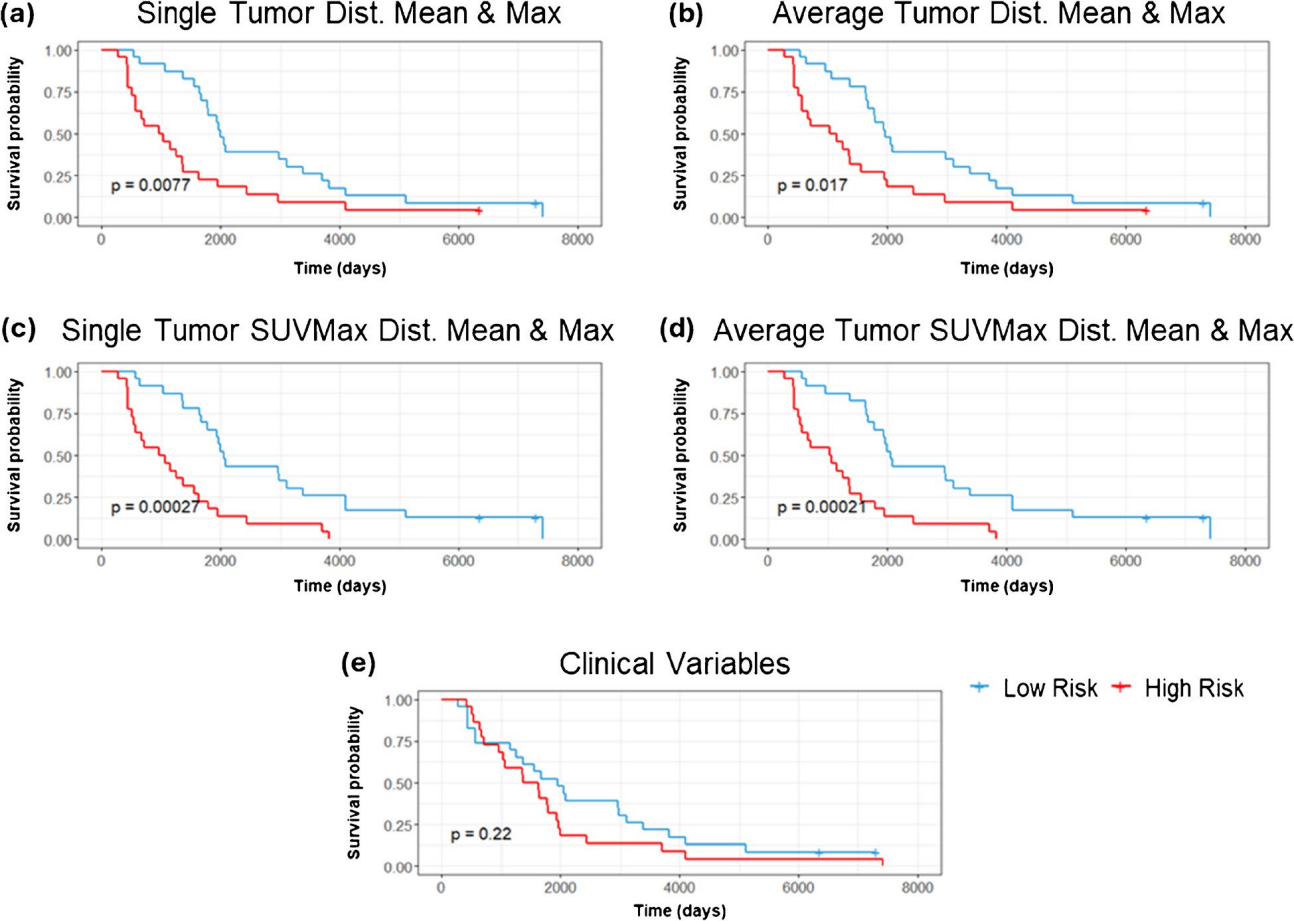
KM-plots of OS for **a** single tumor distance mean and max, **b** average tumor distance mean and max, **c** the combination of significant radiomic variables and SUVmax in the single tumor and **d** average tumor scenarios and the model of OS using **e** all clinical variables for the study cohort (*n = *45). Log-rank *p-*value is displayed in each figure

**Fig. 5:**
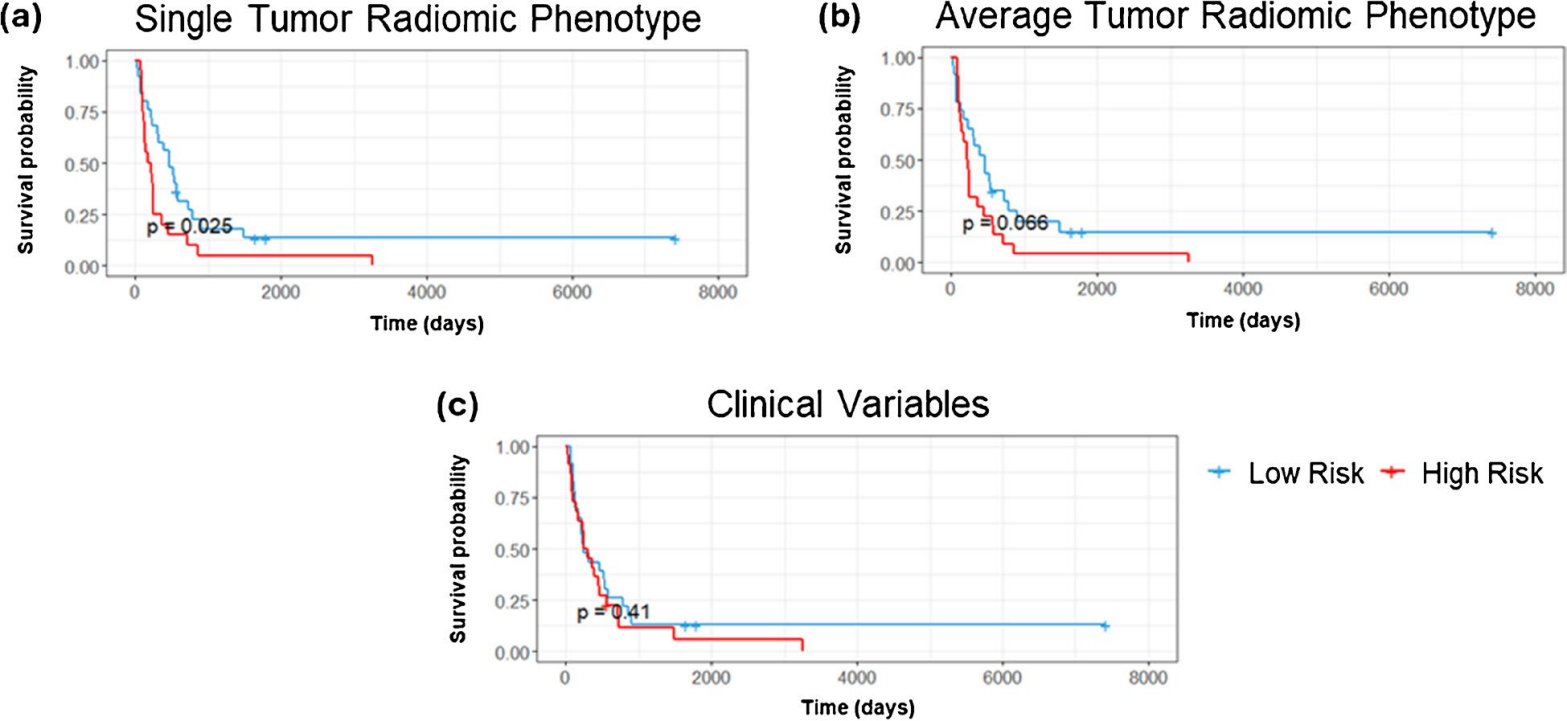
KM-plots of PFS for **a** single tumor and average tumor **b** radiomic phenotype and **c** all clinical variables for the study cohort (*n = *45). Log-rank *p-*value is displayed in each figure

For OS there were 43 events out of the 45 unique patients in this dataset and for PFS there were 41 events out of the 45 patients in this dataset. Depending on the total number of features used, the events per variable (EPV) for most models ranged from 44–11 for OS and 41–10.25 for PFS. The one exception to this EPV range was when we tested all features which resulted in 4.8 EPV for OS and 4.5 EPV for PFS. The results on the study cohort (*n = *45) of the Cox-PH models and KM plots can be seen in Table 1 with results reported by the *C*-statistic and log-rank *p-*value respectively. While the 4D features alone were unable to significantly separate the population into a high or low risk group for OS in the single tumor scenario they were able to do so in the average tumor scenario (C = 0.67, 95% CI: 0.60–0.76, *p* = 0.011). The most significant features of mean and maximum cluster distance were able to significantly separate the population into a high and low risk group for OS considering a single tumor per subject (C = 0.67, 95% CI: 0.55–0.76, *p* = 0.008) (Fig. 4a) and average tumor features (C = 0.65, 95% CI: 0.56–0.74, *p* = 0.017) (Fig. 4b). SUVmax treated as a continuous variable was able to separate the population into a high and low risk group with significance for both the single and average tumor scenario for OS. SUVmax with a binary cutoff at 1.5 was able to separate the cohort into high and low risk groups for OS in the average tumor scenario. Combining the significant radiomic predictors of maximum and mean distance with SUVmax resulted in improved separation of high and low risk groups for the single tumor (*C* = 0.69, 95% CI: 0.63–0.75, *p < *0.005) (Fig. 4c) and average tumor (*C* = 0.69, 95% CI: 0.63–0.77, *p < *0.005) (Fig. 4d) scenarios respectively resulting in the best performing models for OS. All clinical variables alone performed poorly for OS (Fig. 4e) and PFS (Fig. 5c). Combining the most significant predictors from the clinical variables model of HER2 status and histologic subtype with the significant predictors from the 4D features of distance mean and maximum, we achieve better performance than with the clinical predictors alone for OS (*C* = 0.64, 95% CI: 0.56–0.72, *p* = 0.013). Notably, our radiomic phenotype was able to distinguish high and low risk patients with significance for PFS in the single tumor scenario (*C* = 0.67, 95% CI: 0.52–0.83, *p* = 0.025) (Fig. 5a) which no other model was able to do. In our leave one out cross validation models, SUVmax retained prognostic utility for OS in both the single tumor scenario (*C* = 0.61, 95% CI: 0.54–0.69, *p* = 0.047) (Table S5). While the model built on radiomic phenotypes lost significance for PFS predictive ability, using all 4D features as well as SUVmax were significant for PFS in this cross validated setting (*C* = 0.44, 95% CI: 0.36–0.53, *p* = 0.04) and (*C* = 0.5, 95% CI: 0.40–0.59, *p* = 0.047).

**Table 1:** The results of the Cox-PH models and KM plots reported as the *C*-Statistic and log-rank *p-*value respectively

		Overall Survival		Progression Free Survival	
		C-Score	Log-Rank *p-*Value	C-Score	Log-Rank *p-*Value
	**4D Features**	0.68 [0.60, 0.76]	0.082	0.61 [0.51, 0.71]	0.54
	**dist mean, max**	0.67 [0.59, 0.76]	0.008*	0.56 [0.46, 0.66]	0.19
Single Tumor	**Radiomic Phenotype**	0.52 [0.34, 0.70]	0.29	0.67 [0.52, 0.83]	0.025*
	**SUV Max**	0.62 [0.55, 0.70]	0.047*	0.52 [0.44, 0.59]	0.99
	**SUV Max Cutoff 1.5**	0.62 [0.54, 0.70]	0.065	0.62 [0.54, 0.70]	0.75
	**SUV Max, dist mean, max**	0.69 [0.63, 0.75]	< 0.005*	0.56 [0.46, 0.65]	0.16
	**4D Features**	0.67 [0.60, 0.76]	0.011*	0.59 [0.49, 0.69]	0.19
	**dist mean, max**	0.65 [0.56, 0.74]	0.017*	0.58 [0.49, 0.67]	0.11
Tumors Average	**Radiomic Phenotype**	0.50 [0.37, 0.63]	0.53	0.57 [0.46, 0.68]	0.066
	**SUV Max**	0.62 [0.53, 0.71]	0.027*	0.51 [0.43, 0.59]	0.67
	**SUV Max Cutoff 1.5**	0.51 [0.43, 0.59]	0.028*	0.51 [0.43, 0.59]	0.79
	**SUV Max, dist mean, max**	0.69 [0.63, 0.77]	< 0.005*	0.58 [0.50, 0.67]	0.13
Clinical Variables	**All Clinical Variables**	0.56 [0.47, 0.65]	0.22	0.56 [0.46, 0.66]	0.41
	**HER2, Hist., dist mean, max**	0.64 [0.56, 0.72]	0.013*	0.59 [0.52, 0.66]	0.36

In our supplemental analysis of the no-trastuzumab cohort, 3 patients on trastuzumab at the time of imaging were removed; the ability of radiomic phenotype to distinguish high and low risk groups for PFS improved for the single tumor scenario (*C* = 0.69, 95% CI: 0.53–0.86, *p < *0.005) and the average tumor scenario (*C* = 0.62, 95% CI: 0.49–0.75, *p* = 0.044) (supplementary Table S3). In the no-trastuzumab cohort, models that retain significance for OS were the combination of the significant features of distance mean and max and SUVmax in the single tumor (*C* = 0.68, 95% CI: 0.62–0.75, *p < *0.005) and average tumor (*C* = 0.69, 95% CI: 0.61–0.77, *p* = 0.0052) scenarios (supplementary S3). In the no HER2 + breast cancer cohort, a subpopulation of 36 patients where all HER2 positive cases were removed, models combining the significant features of distance mean and max and SUVmax for the single and average tumor scenarios retained significance for OS (supplementary S4). Notably, these models outperformed the models for OS using the clinical variables available in each patient subpopulation we examined. During cross validation in this supplemental analysis SUVmax retains prognostic ability for OS and predictive ability for PFS in both the single tumor and average tumor scenarios (Table S6, Table S7). Additionally, radiomic phenotype, SUVmax with a cutoff at 1.5, and SUVmax combined with significant radiomic predictors were all prognostic for OS in both the no Herceptin and no HER2 + subpopulations in the single tumor and average tumor scenarios.

## Discussion

In this exploratory analysis, we demonstrate the potential for radiomic features to capture the FTH characteristics of tumors in FES PET imaging which can offer enhanced predictive and prognostic power for a cohort with ER positive/HER2 positive and ER positive/HER2 negative metastatic breast cancer patients treated with hormone therapy. The Rad-Fit methodology described is reliant on deterministic and fixed processes such as k-means clustering, Functional Principal Component Analysis. Further, the Markov random field and expectation maximization segmentation refinement approach do not use any stochastic processes meaning the segmentations are deterministic and reproduceable. Though our dataset is limited in size, we attempt to utilize methods such as radiomic phenotype to combat high degree of freedom models and succeed with most models keeping EPV at or above 10 which is generally regarded as the standard practice lower limit for EPV.

Our radiomic phenotype can stratify the population into high and low risk groups for PFS, which suggests that overall tumor heterogeneity is associated with response to hormone therapy, as assessed by PFS, in ER positive/HER2 positive and ER positive/HER2 negative metastatic breast cancer. When tumors exhibit distinct heterogeneous regions, the time activity behavior between clusters is more distinct and thus the resulting clusters are more compact. This distinct time activity behavior between clusters will also bring cluster centers further apart, leading to a decrease in the sum of squared errors (SSE) and an increase in the distance between clusters for tumors with more pronounced heterogeneity. We also see this relationship in our heatmap (Fig. 2) where high distance measures and low SSE measures tend to cluster together under one phenotype indicating more compact clusters and increased distance between clusters of tumors. The other phenotype contains high SSE measures with low distance measures indicating less compact clusters which have cluster centers that are closer together. The high-risk phenotype built on the cohort of 45 ER positive/HER2 positive and ER positive/HER2 negative (phenotype 1 Fig. 2) for PFS is the one that exhibits higher SSE and lower distance, implying worse clustering of subregions within the tumor.

We have identified a subset of radiomic features which alone can identify high and low risk groups for OS in this study cohort, namely the mean and maximum distance between subregions. Combining these radiomic features with standard imaging information, such as SUVmax, further improves our predictive models. Notably, when we remove the ER positive/HER2 positive cases from the initial study cohort of 45 patients (supplementary S4), the combination of significant radiomic features and SUVmax are still able to identify a high and low risk group for OS. Models built from SUVmax on the subset of these radiomic features alone in this reduced cohort could not identify high and low risk groups with significance, indicating complementary information provided by traditional measures of SUVmax and our radiomic features. While the absolute improvements in C-statistics from combining these features were modest, the consistent enhancement in survival curve separation suggests that the 4D radiomic features capture additional biological information not reflected in SUVmax alone. Contrary to our results with the radiomic phenotype, an increase in mean and maximum distance is associated with increased survival time in this cohort. This suggests a poor correlation of PFS and OS outcomes as demonstrated by no significant association of OS and PFS in this cohort by a chi squared test (*p* = 0.12). In our cross-validation analysis, our findings suggest that SUV-based features are relatively robust across validation folds, whereas radiomic features exhibited greater variability in performance. Nonetheless, in our cross-validation analyses excluding patients on Herceptin at time of imaging as well as HER2 positive patients, radiomic phenotypes demonstrated improved prognostic ability for OS, warranting further validation in larger, independent datasets. As SSE was not significantly associated with the outcome of OS, patients that achieve long OS times may have tumors with cluster centers that are far apart but also have very disperse clusters. Given our cohort size and the length of follow up time for OS with the median OS time being 4.7 years, it is difficult to draw strong conclusions of the association of these radiomic features with OS time and a study with a larger population is warranted.

The differing associations between radiomic features and PFS versus OS may reflect the distinct biological and clinical determinants of these endpoints. PFS captures early disease progression, which may be more sensitive to intrinsic tumor aggressiveness and initial therapy response, whereas OS reflects the cumulative influence of subsequent treatments, comorbidities, and other patient-level factors. As such, tumors which progress earlier under endocrine therapy and have shorter PFS remain responsive to subsequent therapies, mitigating their impact on OS.

Moreover, the nature of the heterogeneity itself may be relevant. In the Rad-Fit method, the expectation maximization algorithm estimates voxel cluster membership prior probabilities from FPCs, a factor in these probabilities is the cluster assignment of neighboring voxels. When cluster boundaries are broad and intermixed, the higher proportion of neighboring voxels from other clusters can increase the SSE of individual clusters and reduce the measured distance between cluster centers. This morphological dimension of heterogeneity may influence the clustering metrics differently for PFS and OS, suggesting that both the extent and spatial organization of kinetic heterogeneity could be biologically and prognostically important.

Compared to our initial study of dynamic FDG PET in patients undergoing neoadjuvant chemotherapy that included three subtype of breast cancer (ER positive/HER2 negative, HER2 positive, and triple-negative breast cancer) [58], this study of dynamic FES PET in patients treated with endocrine therapy included only patients with either ER positive/HER2 negative or ER positive/HER2 positive tumors. Our approach does not assume a set number of clusters that should be seen across all tumors; thus, a list of cluster distances and SSE values cannot be used as a feature vector since the length of the vector would change with the number of clusters. We summarize the between cluster distance and within cluster SSE with the mean, minimum, maximum, and standard deviation. We used the scaled silhouette score to determine the optimal number of clusters scaled by the difference in size of largest and smallest cluster as clusters with few voxels are considered poor clusterings. All singleton clusterings are rejected for the same reason. The example cases shown in Fig. 3 and supplementary Fig. S2 demonstrate the ability of our Rad-Fit approach to effectively utilize the FPCs of each voxel in the tumor to cluster subregions of unique activity which are then reflected in the summarized time activity curve of each region (Fig. 3b & supplementary Fig. S2b). These cases demonstrate how an optimal cluster number selection approach via the silhouette score can potentially identify heterogeneous tumor subregions.

The SSE of each subregion was another means to measure within cluster similarity which may explain why the two distance metrics of average and maximum distance were the most significant factors in determining the risk of individuals OS according to the 4D radiomic features. While the SSE has been somewhat optimized via the silhouette score across all tumors, it is possible to have two tumors which have well clustered subregions, however the subregions themselves may either be close or very separated when examined in FPC space (supplementary Fig. S5).

There is an imbalance in the distribution of HER2 positive cases between the two radiomic phenotypes with seven out of nine cases belonging to one phenotype and the other two to the other phenotype with a near significant distribution between these two groups (*p* = 0.057) by a Fisher’s exact test. Furthermore, the histogram (supplementary Fig. S6) of mean cluster distance separated by patients who are positive for HER2 shows that the HER2 positive patients span the range of values for the mean distance feature and HER2 status did not separate this radiomic feature significantly into two distinct groups using a Welch’s t-test (*p* = 0.12). The limited number of HER2 positive patients in our cohort prevents us from drawing definitive conclusions about the association between our radiomic features and this specific breast cancer subtype. The potential for dynamic radiomic features to capture properties of cancers that may indicate an ER positive/HER2 positive like phenotype merits further study. The mechanistic basis for our observations is rooted in the FES uptake dynamics which reflect estrogen receptor binding and tracer clearance. Any heterogeneity in these processes may indicate differences in tumor biology and endocrine sensitivity. Given our limited sample size, the possibility of chance findings cannot be excluded. We are encouraged by the uncovered hypothesis-generating signatures. Future clinical utility will depend on validation in larger prospectively collected datasets. While there are likely other features that may be of significance, we focus on relatively sparse models to complement our dataset size. Limitations imposed by dataset size do not allow us to have distinct model development and testing datasets, though we did implement a cross-validation framework for internal validation. In addition, we acknowledge that these historical studies were performed on a PET-only scanner two decades ago with a limited field of view and reduced image quality that do not reflect the capabilities of modern PET/CT devices. Another limitation of this study is that ER positive breast cancer patients included in this study were treated with endocrine therapy alone and not endocrine therapy plus a CDK4/6, which is now the standard of care. These factors limit the direct applicability of our findings to current treatment paradigms. Despite these limitations, this dataset does offer a unique experimental perspective for assessing tracer kinetics which is precisely the type of information the Rad-Fit method leverages. The observed trends are promising and support the need for a prospective study with a larger cohort.

## Conclusion

In this exploratory study, we show that 4D radiomic features extracted from dynamic FES PET images using the established Rad-Fit method can significantly predict risk and outcomes in metastatic ER positive breast cancer. The radiomic phenotype which describes overall tumor heterogeneity is the best predictor for PFS in this cohort and individual metrics of the distinctness of tumor subregion time activity such as tumor subregion distance in functional principal component space appear to perform as the best radiomic predictors for risk stratification of OS. Combination of the significant radiomic features of distance mean and maximum with other clinical variables such as HER2 status and SUVmax augment and improve risk stratification. The prognostic model built from the significant radiomic features with SUVmax in the absence of any HER2 + cases remained significant for OS, even when the model built from clinical variables alone did not perform well, indicating the addition of information from the radiomic features is complementary to the clinical information.

## Supplementary Information

Below is the link to the electronic supplementary material.

Supplementary file1 (DOCX 443 KB)

## Data Availability

The datasets generated during and/or analyzed during the current study are available from the corresponding author on reasonable request.
