# Supplementary material for: Novel 4D radiomics applied to dynamic FES PET images to improve prediction of breast cancer response to ER-targeted therapy
Source: Eur J Nucl Med Mol Imaging. 2025 Nov 23;53(5):3128–39. doi: 10.1007/s00259-025-07570-y (PMC13013131; doi:10.1007/s00259-025-07570-y)
Supplement: Supplementary file 1 — Supplementary file1 (DOCX 443 KB) [file 259_2025_7570_MOESM1_ESM.docx]

**Table S1**. Table of associated clinical variables for the study cohort

| Variable | Category | Number of Patients (n=45) |
| --- | --- | --- |
| Age | Mean | 55.72 |
|  | Median | 55.9 |
|  | Range [min, max] | [35, 76.7] |
|  |  |  |
| HER2 IHC | Positive | 9 |
|  | Negative | 36 |
|  |  |  |
| Blood Estradiol | <20 | 31 |
|  | ≥20 | 14 |
|  |  |  |
| ER Status | Positive | 38 |
|  |  |  |
|  | Positive | 32 |
| PR Status | Negative | 4 |
|  | Unknown | 2 |
|  |  |  |
| Menopausal Status | Premenopausal | 6 |
|  | Postmenopausal | 39 |
|  |  |  |
| Pre-FES Therapy | Prior Chemo [yes, no] | [37, 8] |
|  | Prior Radiation [yes, no] | [25, 20] |
|  | Prior Hormone Therapy [yes, no] | [31, 14] |
|  |  |  |
| Post-FES Therapy | Letrozole | 19 |
|  | Exemestane | 4 |
|  | Anastrozole | 9 |
|  | Tamoxifen | 5 |
|  | Herceptin & Other Agents | 3 |
|  | Fluvestrant & Other Agents | 5 |


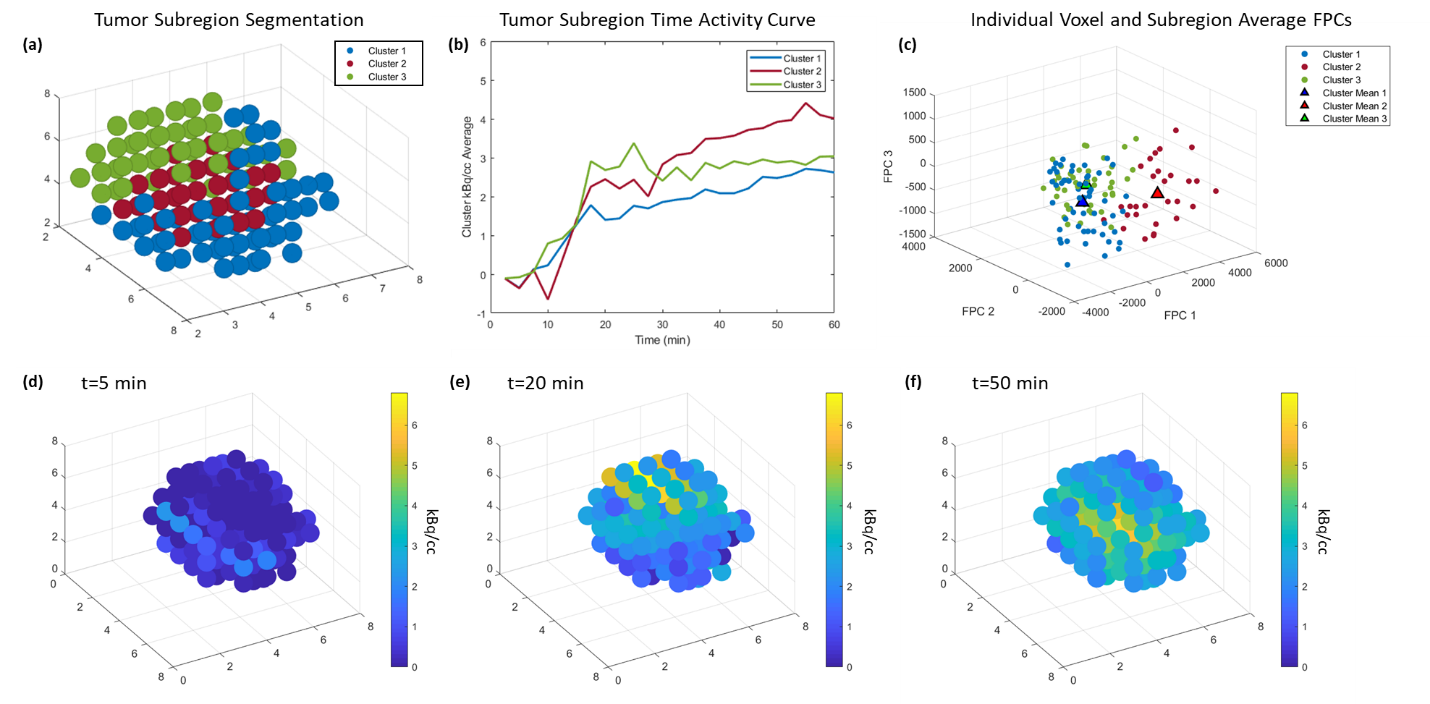
**Fig S2**. **(a)** An example segmentation of a tumor into three distinct subregions and **(b)** the average intensity from each subregion. **(c)** Each voxel from each subregion is shown according to their first three FPCs with the centers of each cluster of points also shown. Tumor region intensity is shown at **(d)** 5 minutes is homogeneous, **(e)** at 20 minutes parts of the upper subregion hare reaching a higher intensity than the other two, **(f)** at 50 minutes the subregion which comprises the core of the tumor demonstrates increased intensity as that region has continued to aggregate radiotracer

**Table S3**. Table of the Cox-PH models and KM plots reported as the C-Statistic and log-rank p-value respectively on the cohort of 42 patients where 3 patients with ER+/HER2+ breast cancer receiving trastuzumab at the time of imaging were removed.

|  |  | **Overall Survival** | | **Progression Free Survival** | |
| --- | --- | --- | --- | --- | --- |
|  |  | **C-Score** | **Log-Rank p-Value** | **C-Score** | **Log-Rank p-Value** |
| **Single Tumor** | **4D Features** | 0.66 [0.57, 0.76] | 0.14 | 0.60 [0.49, 0.70] | 0.29 |
|  | **dist mean, max** | 0.64 [0.55, 0.73] | 0.075 | 0.54 [0.43, 0.65] | 0.22 |
|  | **Radiomic Phenotype** | 0.44 [0.26, 0.61] | 0.83 | 0.69 [0.53, 0.86] | <0.005* |
|  | **SUVmax** | 0.65 [0.58, 0.72] | 0.04* | 0.52 [0.44, 0.60] | 0.64 |
|  | **SUVmax Cutoff 1.5** | 0.65 [0.58, 0.72] | 0.017* | 0.65 [0.58, 0.72] | 0.60 |
|  | **SUVmax, dist mean, max** | 0.68 [0.62, 0.75] | <0.005* | 0.54 [0.43, 0.64] | 0.22 |
| **Tumors Average** | **4D Features** | 0.65 [0.56, 0.75] | 0.03* | 0.59 [0.49, 0.68] | 0.25 |
|  | **dist mean, max** | 0.60 [0.51, 0.70] | 0.71 | 0.58 [0.48, 0.68] | 0.17 |
|  | **Radiomic Phenotype** | 0.43 [0.30, 0.56] | 0.91 | 0.62 [0.49, 0.75] | 0.044* |
|  | **SUVmax** | 0.66 [0.58, 0.74] | 0.036* | 0.51 [0.43, 0.60] | 0.80 |
|  | **SUVmax Cutoff 1.5** | 0.51 [0.43, 0.60] | 0.005* | 0.51 [0.43, 0.60] | 0.71 |
|  | **SUVmax, dist mean, max** | 0.69 [0.61, 0.77] | 0.0052* | 0.57 [0.49, 0.65] | 0.17 |
| **Clinical Variables** | **All Clinical Variables** | 0.52 [0.43, 0.62] | 0.52 | 0.58 [0.49, 0.66] | 0.20 |
|  | **HER2, Hist., dist mean, max** | 0.61 [0.53, 0.69] | 0.081 | 0.58 [0.50, 0.66] | 0.39 |

**Table S4**. Table of the Cox-PH models and KM plots reported as the C-Statistic and log-rank p-value respectively on the cohort of 36 patients where an additional 6 patients that had ER+/HER2+ disease were removed

|  |  | **Overall Survival** | | **Progression Free Survival** | |
| --- | --- | --- | --- | --- | --- |
|  |  | **C-Score** | **Log-Rank p-Value** | **C-Score** | **Log-Rank p-Value** |
| **Single Tumor** | **4D Features** | 0.68 [0.60, 0.77] | 0.86 | 0.61 [0.51, 0.71] | 0.31 |
|  | **dist mean, max** | 0.63 [0.53, 0.72] | 0.51 | 0.56 [0.45, 0.67] | 0.31 |
|  | **Radiomic Phenotype** | 0.53 [0.35, 0.71] | 0.67 | 0.54 [0.34, 0.74] | 0.18 |
|  | **SUVmax** | 0.63 [0.55, 0.71] | 0.068 | 0.53 [0.44, 0.61] | 0.41 |
|  | **SUVmax Cutoff 1.5** | 0.63 [0.55, 0.71] | 0.088 | 0.37 [0.29, 0.45] | 0.88 |
|  | **SUVmax, dist mean, max** | 0.65 [0.57, 0.72] | 0.021* | 0.57 [0.48, 0.65] | 0.24 |
| **Tumors Average** | **4D Features** | 0.69 [0.59, 0.79] | 0.0086* | 0.57 [0.46, 0.69] | 0.58 |
|  | **dist mean, max** | 0.63 [0.52, 0.73] | 0.23 | 0.60 [0.50, 0.70] | 0.23 |
|  | **Radiomic Phenotype** | 0.51 [0.38, 0.65] | 0.65 | 0.56 [0.44, 0.68] | 0.22 |
|  | **SUVmax** | 0.63 [0.54, 0.71] | 0.19 | 0.54 [0.46, 0.62] | 0.34 |
|  | **SUVmax Cutoff 1.5** | 0.46 [0.38, 0.54] | 0.028* | 0.54 [0.46, 0.62] | 0.69 |
|  | **SUVmax, dist mean, max** | 0.66 [0.57, 0.75] | 0.026* | 0.61 [0.52, 0.69] | 0.11 |
| **Clinical Variables** | **All Clinical Variables** | 0.55 [0.45, 0.66] | 0.58 | 0.57 [0.46, 0.67] | 0.20 |
|  | **Hist., dist mean, max** | 0.61 [0.52, 0.70] | 0.07 | 0.58 [0.50, 0.66] | 0.77 |

**Table S5**. Table of the Cox-PH models and KM plots reported as the C-Statistic and log-rank p-value respectively on the cohort of 45 patients. Results are 5 fold cross validated.

|  |  | **Overall Survival** | | **Progression Free Survival** | |
| --- | --- | --- | --- | --- | --- |
|  |  | **C-Score** | **Log-Rank p-Value** | **C-Score** | **Log-Rank p-Value** |
| **Single Tumor** | **4D Features** | 0.59 [0.50, 0.68] | 0.27 | 0.48 [0.40, 0.57] | 0.71 |
|  | **dist mean, max** | 0.57 [0.47, 0.65] | 0.58 | 0.50 [0.41, 0.59] | 0.21 |
|  | **Radiomic Phenotype** | 0.41 [0.29, 0.54] | 0.60 | 0.55 [0.47, 0.64] | 0.07 |
|  | **SUVmax** | 0.61 [0.54, 0.69] | 0.047* | 0.50 [0.41, 0.58] | 0.06 |
|  | **SUVmax Cutoff 1.5** | 0.46 [0.39, 0.54] | 0.065 | 0.38 [0.28, 0.49] | 0.75 |
|  | **SUVmax, dist mean, max** | 0.62 [0.54, 0.70] | <0.005* | 0.49 [0.40, 0.59] | 0.41 |
| **Tumors Average** | **4D Features** | 0.58 [0.49, 0.67] | 0.20 | 0.44 [0.36, 0.53] | 0.04* |
|  | **dist mean, max** | 0.56 [0.46, 0.66] | 0.58 | 0.54 [0.45, 0.62] | 0.22 |
|  | **Radiomic Phenotype** | 0.47 [0.36, 0.58] | 0.36 | 0.52 [0.44, 0.61] | 0.27 |
|  | **SUVmax** | 0.60 [0.52, 0.69] | 0.07 | 0.50 [0.40, 0.59] | 0.023* |
|  | **SUVmax Cutoff 1.5** | 0.52 [0.46, 0.59] | 0.028* | 0.38 [0.27, 0.49] | 0.79 |
|  | **SUVmax, dist mean, max** | 0.64 [0.57, 0.71] | <0.005* | 0.53 [0.44, 0.61] | 0.43 |
| **Clinical Variables** | **All Clinical Variables** | 0.46 [0.38, 0.56] | 0.77 | 0.30 [0.22, 0.38] | 0.25 |
|  | **Hist., dist mean, max** | 0.52 [0.41, 0.64] | 0.65 | 0.48 [0.39, 0.58] | 0.68 |

**Table S6**. Table of the Cox-PH models and KM plots reported as the C-Statistic and log-rank p-value respectively on the cohort of 42 patients where 3 patients with ER+/HER2+ breast cancer receiving trastuzumab at the time of imaging were removed. Results are 5 fold cross validated.

|  |  | **Overall Survival** | | **Progression Free Survival** | |
| --- | --- | --- | --- | --- | --- |
|  |  | **C-Score** | **Log-Rank p-Value** | **C-Score** | **Log-Rank p-Value** |
| **Single Tumor** | **4D Features** | 0.57 [0.48, 0.67] | 0.8 | 0.44 [0.34, 0.55] | 0.35 |
|  | **dist mean, max** | 0.60 [0.49, 0.70] | 0.24 | 0.42 [0.34, 0.51] | 0.63 |
|  | **Radiomic Phenotype** | 0.32 [0.23, 0.41] | 0.008* | 0.56 [0.46, 0.67] | 0.83 |
|  | **SUVmax** | 0.65 [0.58, 0.72] | 0.04* | 0.43 [0.34, 0.52] | 0.02* |
|  | **SUVmax Cutoff 1.5** | 0.49 [0.42, 0.55] | 0.017* | 0.39 [0.28, 0.51] | 0.60 |
|  | **SUVmax, dist mean, max** | 0.65 [0.58, 0.71] | 0.01* | 0.42 [0.33, 0.51] | 0.70 |
| **Tumors Average** | **4D Features** | 0.55 [0.46, 0.65] | 0.59 | 0.38 [0.30, 0.47] | 0.75 |
|  | **dist mean, max** | 0.58 [0.48, 0.69] | 0.55 | 0.47 [0.38, 0.55] | 0.58 |
|  | **Radiomic Phenotype** | 0.34 [0.25, 0.43] | 0.01* | 0.57 [0.47, 0.67] | 0.79 |
|  | **SUVmax** | 0.66 [0.58, 0.74] | <0.005* | 0.47 [0.37, 0.56] | 0.01* |
|  | **SUVmax Cutoff 1.5** | 0.58 [0.51, 0.65] | 0.005* | 0.38 [0.27, 0.50] | 0.71 |
|  | **SUVmax, dist mean, max** | 0.65 [0.59, 0.73] | 0.01* | 0.48 [0.39, 0.56] | 0.52 |
| **Clinical Variables** | **All Clinical Variables** | 0.34 [0.25, 0.43] | <0.005* | 0.41 [0.33, 0.50] | 0.33 |
|  | **Hist., dist mean, max** | 0.47 [0.36, 0.59] | 0.56 | 0.42 [0.36, 0.49] | 0.36 |

**Table S7**. Table of the Cox-PH models and KM plots reported as the C-Statistic and log-rank p-value respectively on the cohort of 36 patients where an additional 6 patients that had ER+/HER2+ disease were removed. Results are 5 fold cross validated.

|  |  | **Overall Survival** | | **Progression Free Survival** | |
| --- | --- | --- | --- | --- | --- |
|  |  | **C-Score** | **Log-Rank p-Value** | **C-Score** | **Log-Rank p-Value** |
| **Single Tumor** | **4D Features** | 0.58 [0.48, 0.67] | 0.80 | 0.45 [0.34, 0.55] | 0.36 |
|  | **dist mean, max** | 0.60 [0.49, 0.70] | 0.24 | 0.42 [0.34, 0.51] | 0.63 |
|  | **Radiomic Phenotype** | 0.32 [0.23, 0.41] | 0.008* | 0.56 [0.46, 0.67] | 0.83 |
|  | **SUVmax** | 0.65 [0.58, 0.72] | 0.04* | 0.43 [0.34, 0.52] | 0.02* |
|  | **SUVmax Cutoff 1.5** | 0.49 [0.42, 0.55] | 0.017* | 0.39 [0.28, 0.51] | 0.6 |
|  | **SUVmax, dist mean, max** | 0.65 [0.58, 0.71] | 0.01* | 0.42 [0.33, 0.51] | 0.70 |
| **Tumors Average** | **4D Features** | 0.55 [0.46, 0.65] | 0.59 | 0.38 [0.30, 0.47] | 0.74 |
|  | **dist mean, max** | 0.58 [0.48, 0.69] | 0.55 | 0.47 [0.38, 0.55] | 0.58 |
|  | **Radiomic Phenotype** | 0.34 [0.24, 0.43] | 0.01 | 0.57 [0.47, 0.67] | 0.79 |
|  | **SUVmax** | 0.66 [0.58, 0.74] | <0.005* | 0.47 [0.37, 0.56] | 0.01* |
|  | **SUVmax Cutoff 1.5** | 0.58 [0.51, 0.65] | 0.005* | 0.39 [0.27, 0.50] | 0.71 |
|  | **SUVmax, dist mean, max** | 0.65 [0.58, 0.73] | 0.01* | 0.48 [0.39, 0.56] | 0.51 |
| **Clinical Variables** | **All Clinical Variables** | 0.34 [0.25, 0.43] | 0.001* | 0.41 [0.33, 0.50] | 0.33 |
|  | **Hist., dist mean, max** | 0.47 [0.36, 0.59] | 0.56 | 0.42 [0.36, 0.49] | 0.36 |

**Table S8**. Table of hazard ratio, 95% confidence interval, and Wald test p value for each model variable in the study cohort of 45 patients.

**Table S9**. Table of hazard ratio, 95% confidence interval, and Wald test p value for each model variable in the study cohort of 42 patients where those patients on trastuzumab at time of imaging have been removed.

**Table S10**. Table of hazard ratio, 95% confidence interval, and Wald test p value for each model variable in the study cohort of 36 patients where those patients that are HER2+ have been removed.

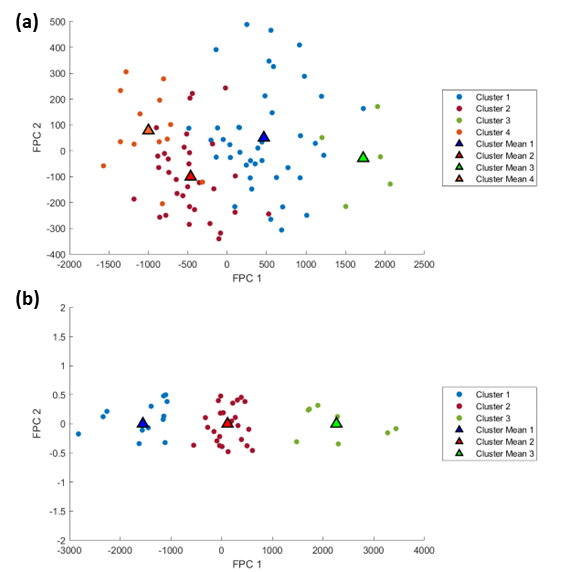


**Fig. S5** Voxel locations and cluster centers in the first two FPC dimensions for two tumors which had similar average SSE of **(a)** 0.12 and **(b)** 0.13 indicating similar clustering performance but differed in an order of magnitude in separation of cluster centers of **(a)** 13277 and **(b)** 2742


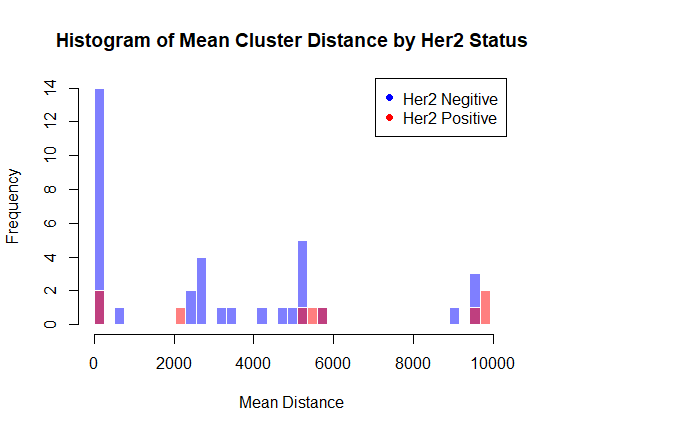


**Fig. S6** Histogram of the radiomic features of mean distance separated into patients who were HER2 positive or negative (n=45). The HER2 positive cases are distributed throughout the range of mean distance values indicating that our 4D radiomic features are not directly mimicking known biological predictors
